# Real-World Weekly Efficacy Analysis of Faricimab in Patients with Age-Related Macular Degeneration

**DOI:** 10.3390/bioengineering11050478

**Published:** 2024-05-10

**Authors:** Daniel R. Muth, Katrin F. Fasler, Anders Kvanta, Magdalena Rejdak, Frank Blaser, Sandrine A. Zweifel

**Affiliations:** 1 Department of Ophthalmology, University Hospital Zurich, University of Zurich, 8091 Zurich, Switzerland; 2 Division of Eye and Vision, Department of Clinical Neuroscience, Karolinska Institutet, 171 77 Stockholm, Sweden; anders.kvanta@ki.se; 3 St. Erik Eye Hospital (S:t Eriks Ögonsjukhus), 171 64 Solna, Sweden

**Keywords:** age-related macular degeneration, AMD, intravitreal injection, IVT, IVI, anti-VEGF, neovascular, exudative, MNV, CNV, faricimab, real-world data, RWD, OCT

## Abstract

**Objectives**: This study entailed a weekly analysis of real-world data (RWD) on the safety and efficacy of intravitreal (IVT) faricimab in neovascular age-related macular degeneration (nAMD). **Methods**: A retrospective, single-centre clinical trial was conducted at the Department of Ophthalmology, University Hospital Zurich, University of Zurich, Switzerland, approved by the Cantonal Ethics Committee of Zurich, Switzerland. Patients with nAMD were included. Data from patient charts and imaging were analysed. The safety and efficacy of the first faricimab injection were evaluated weekly until 4 weeks after injection. **Results**: Sixty-three eyes with a complete 4-week follow-up were enrolled. Six eyes were treatment-naïve; fifty-seven eyes were switched to faricimab from another treatment. Neither group showed signs of retinal vasculitis during the 4 weeks after injection. Central subfield thickness (CST) and volume (CSV) showed a statistically significant decrease compared to the baseline in the switched group (CST: *p* = 0.00383; CSV: *p* = 0.00702) after 4 weeks. The corrected visual acuity returned to the baseline level in both groups. The macular neovascularization area decreased in both groups, but this was not statistically significant. A complete resolution of sub- and intraretinal fluid after 4 weeks was found in 40% (switched) and 75% (naïve) of the treated patients. **Conclusions**: The weekly follow-ups reflect the structure–function relationship beginning with a fast functional improvement within two weeks after injection followed by a return to near-baseline levels after week 3. The first faricimab injection in our cohort showed a high safety profile and a statistically significant reduction in macular oedema in switched nAMD patients.

## 1. Introduction

Vascular endothelial growth factor (VEGF) is one of the most studied angiogenesis-inducing factors of recent years. Its existence and its effects on the retina were first assumed by I.C. Michaelson in 1948 [1]. With it not being possible to isolate and sequence the factor at that time, Michaelson called it “Factor X” [1]. Twenty-seven years later, the factor was called “vascular permeability factor” [2,3] (VPF) by Gilbert Lagrue and co-workers in 1975 as it was considered to be systemically responsible for vascular leakage in association with nephrotic syndrome. A partial isolation of VPF was achieved in 1983 by Donald R. Senger and co-workers [2,4]. In 1989, David W. Leung and co-workers discovered and isolated a factor they called “vascular endothelial growth factor” (VEGF) [5]. They believed that they had found an additional factor to induce angiogenesis with resulting vascular permeability. In the same year, 1989, having DNA-sequenced both factors, it turned out that VPF and VEGF were identical [4,5,6,7]. Consecutively, “VEGF” became the main utilised term in the literature.

Angiogenesis is induced in the moment when the net balance of inducing factors outweighs inhibiting factors, the so-called “angiogenic switch” [8,9].

In 1993, the first monoclonal antibodies against VEGF were developed and showed efficacy in the in vivo inhibition of tumour angiogenesis [10,11]. In 1994, elevated vitreous and aqueous humour VEGF levels up to 3–10 ng/mL were detected in the presence of active proliferative retinal diseases such as diabetic retinopathy, central retinal vein occlusion, retinopathy of prematurity, and neovascular age-related macular degeneration (nAMD) [12,13]. Expression of VEGF mRNA and protein in surgically removed MNV lesions was soon demonstrated, providing supportive evidence linking VEGF to nAMD pathogenesis [14]. It was further shown that tissue ischaemia induced the upregulation of VEGF that in turn correlated with neovascularization [15,16]. These discoveries in 1997 led to the development of the first humanised antiangiogenic drug binding to VEGF-A, the principal angiogenic VEGF isoform [17]. It was initially approved by the American Food and Drug Association (FDA) in 2004 for the treatment of colon cancer and was a milestone in oncologic therapy [13]. The same year, the first intraocular anti-VEGF, pegaptanib, was FDA-approved for the treatment of neovascular AMD (nAMD) [13]. It selectively bound the VEGF_165_ splice variant of VEGF-A [18]. Ranibizumab 0.5 mg (binding VEGF-A [18]) was approved for the treatment of nAMD 2 years later, in 2006 [19]. Aflibercept 2 mg, a chimeric fusion protein binding VEGF-A, VEGF-B, and placental growth factor (PIGF), followed with FDA approval in 2011 [20].

Intravitreal anti-VEGF treatment enabled, for the first time, improvements in anatomical structure and function (best-corrected visual acuity) in nAMD to values better than baseline. However, the positive treatment effect comes with a significant treatment burden for the patient, healthcare providers, and the public health system. Therefore, efforts have been made to prolong the treatment effect of intravitreal injection (IVT, synonymously IVI). Higher-dosed drugs were evaluated, such as ranibizumab 2 mg (LAST study [21]; SAVE study [22,23]) and aflibercept 8 mg (PULSAR study [24]), or drugs with a higher equivalent molar dose, such as brolucizumab 6 mg (FDA approval: 2019 [25]). The latest addendum is faricimab (FDA approval: January 2022 [26]; SWISSMEDIC approval: May 2022 [27]; European Medicines Agency, EMA: September 2022 [28]), the first intraocular approved bispecific antibody targeting VEGF-A and angiopoetin-2 (Ang-2). By inhibiting two pathways that are associated with macular neovascularisation (MNV), the interval between treatment injections could be prolonged as the results of phase-3 trials suggested (TENAYA trial; LUCERNE trial [29]). The presented study therefore evaluates the reproducibility of the safety and efficacy of faricimab in real-world conditions.

## 2. Materials and Methods

### 2.1. Ethics

Ethics Committee approval was obtained from the local Ethics Committee of the Canton of Zurich, Switzerland (project no. PB_2016_00264). This study adheres to the tenets of the 1964 Declaration of Helsinki and its later amendments.

### 2.2. Study Design

This is a retrospective, single-centre clinical trial conducted at the Department of Ophthalmology, University Hospital Zurich, University of Zurich, Switzerland.

### 2.3. Data Collection

Clinical patient data were extracted from the electronic patient chart system (KISIM, CISTEC AG, Zurich, Switzerland) and the imaging viewers Heidelberg Eye Explorer (Heidelberg Engineering, Heidelberg, Germany), Nikon Optos Viewer (Optos Inc., Marlborough, MA, USA), Solix Viewer (Visionix International SAS, Pont-de-l’Arche, France), and Zeiss Plex Elite Viewer (Carl Zeiss Meditec AG, Jena, Germany). Patients with nAMD were enrolled. Previous treatment with intravitreal anti-VEGF drugs, i.e., ranibizumab, aflibercept, and bevacizumab, was not an exclusion criterion, but the interval from the last injection had to be at least 4 weeks. Patients with previous intravitreal brolucizumab or steroid injections, para-/retrobulbar steroid injections, previous macular laser treatment, photodynamic therapy (PDT), or pars-plana vitrectomy were excluded from the study. Data were reviewed before the first IVT of faricimab was given (baseline) and weekly after the first faricimab IVT for the following 4–5 weeks until the second faricimab IVT was administered. Corrected visual acuity (CVA) with auto-refraction (Nidek NT-530/510, Nidek Company, Ltd., Hirioshi-cho, Japan) or current glasses was tested according to the Early Treatment of Diabetic Retinopathy Study (ETDRS) scheme; intraocular pressure (IOP) was taken by air-puff tonometry (Nidek Company, Ltd., Hirioshi-cho, Japan) or Goldman applanation tonometry. Further clinical data on drug safety were extracted from the patient charts: anterior chamber (AC) cells within a 1 mm × 1 mm slit beam field and AC flare as defined by the Standardization of Uveitis Nomenclature (SUN) Working Group [30]. Vitreous cells were assessed clinically in mydriasis at the slit lamp within a 1 mm × 1 mm slit beam field using a 78D, 66D, or Digital Widefield lens (Volk Optical, Mentor, OH, USA). The retinal vessel status was assessed clinically based on vessel perfusion, vessel calliper, tortuosity, and bleedings.

### 2.4. Image Analysis

**Figure 1:**
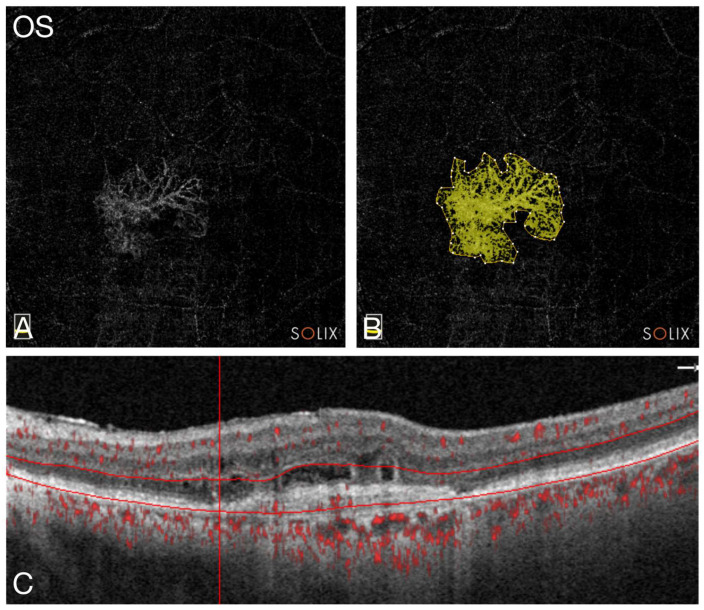
(**A**) En-face angiography compilation of the “Outer Retina” slab of optical coherence tomography angiography (OCTA) scan with macular neovascularisation (MNV) (network of white lines). (**B**) Same en-face OCTA image as in A but with marked outer boundary of MNV lesion (yellow line with white dots) and automatically detected vascular network (network of yellow undotted lines) by built-in software algorithm. (**C**) Corresponding cross-sectional OCT B-scan with visible retinal pigment epithelium (RPE) detachment (PED) and double-layer sign (DLS) between RPE and underlying Bruch’s membrane (BM). Horizontal red lines mark the boundaries of “Outer Retina” slab. The anatomical layers within the slab are compiled to the en-face angiography image. Red dotted overlays indicate areas with detected blood flow.

Imaging was carried out by structural spectral-domain optical coherence tomography (SD-OCT) (Spectralis, Heidelberg Engineering, Heidelberg, Germany), using two OCT angiography (OCTA) devices (Solix, Visionix International SAS, Pont-de-l’Arche, France; Plex Elite, Carl Zeiss Meditec AG, Jena, Germany), based on spectral-domain (Solix) and swept-source OCT (SS-OCT) technology (Plex Elite). Fundus imaging was carried out with a false-colour ultra-widefield camera (California, Optos Inc., Marlborough, MA, USA). Image quality and foveal centration of the ETDRS grid, which was introduced by the ETDRS Research Group (report 10 [31]), was reviewed on all OCT scans. Manual corrections were carried out if necessary. As a structural–anatomical correlate for treatment efficacy, the thickness (CST) and the volume (CSV) central subfield of the central 1-millimetre circle of the ETDRS grid was measured in micrometres [µm] and cubic millimetres [mm^3^]. On the OCTA images by the Solix device, the MNV flow area was measured using the built-in software. Therefore, the “outer retina” preset slab was selected. If detectable, the outer border (perimeter) of the MNV lesion was manually marked on the en-face reconstruction of the slab (Figure 1A,B) in correlation with the cross-sectional image (Figure 1C).

The Solix software then automatically detected the MNV network and calculated the MNV area in square millimetres [mm^2^]. The Consensus on Neovascular AMD Nomenclature (CONAN) classification was used to characterise the MNV type based on the OCTA images [32,33]: (1) MNV type 1: vessels confined to the sub-retinal pigment epithelium (sub-RPE) space; (2) MNV type 2: vessels proliferating above the RPE in the sub-neurosensory, subretinal space, or mixed type 1/2 MNV; and (3) MNV type 3: vessels of intraretinal origin or any combined type 3 MNV.

### 2.5. Statistical Analysis

Data were organised in Excel (Microsoft Corp., Redmond, WA, USA) and statistically analysed using RStudio (RStudio PBC, Boston, MA, USA) and StatPlus:mac (AnalystSoft, Walnut, CA, USA). The statistical significance level (*α*) was defined as 0.05 for all the tests used. The results of the statistical analyses with a *p*-value less than 0.05 (*p* < 0.05) were interpreted as statistically significant. Descriptive statistics such as the arithmetic mean with standard deviation (±SD), 95% confidence interval (95%CI), and median with quartile ranges (Q1–Q3) and minimum–maximum (min–max) range were computed. The data were analysed regarding normal distribution using the Kolmogorov–Smirnov and Shapiro–Wilk tests. For normally distributed variables, differences between baseline prior to the first faricimab IVT and 4 weeks after the first faricimab IVT were calculated using a one-sample, dependent, 2-tailed *t*-test. For non-normally distributed variables, a two-tailed Wilcoxon signed-rank test was applied.

## 3. Results

### 3.1. Demographics

Sixty-three eyes (*n* = 63) of 53 AMD patients with a completed 4-week follow-up were enrolled in this study. Six of those eyes (five patients) had been treatment-naïve at the time of their first faricimab injection and were therefore allocated to a separate treatment-naïve subgroup. The other 57 eyes (48 patients) had been switched to faricimab from another anti-VEGF IVT and were therefore allocated to the switched subgroup. The previous median injection interval was 4 weeks (interquartile range (Q1–Q3): 4–5 weeks). The median number of anti-VEGF IVTs prior to faricimab was 33 (min–max range: 5–83). Detailed demographic data are listed in Table 1.

### 3.2. Safety

Concerning clinical safety data, the switched group showed a statistically but not clinically significant difference in IOP at the 4-week FU compared to the baseline (Table 2). The treatment-naïve group did not show statistically significant differences in IOP and no signs of intraocular inflammation or vasculitis (Table 2). The switched group presented with two cases with mild AC cells (0.5+ to 1+) at follow-up weeks 2 and 3, respectively (Table 2). These findings were clinically judged as non-significant. Therefore, the further treatment regime was not changed.

### 3.3. Efficacy

**Figure 2:**
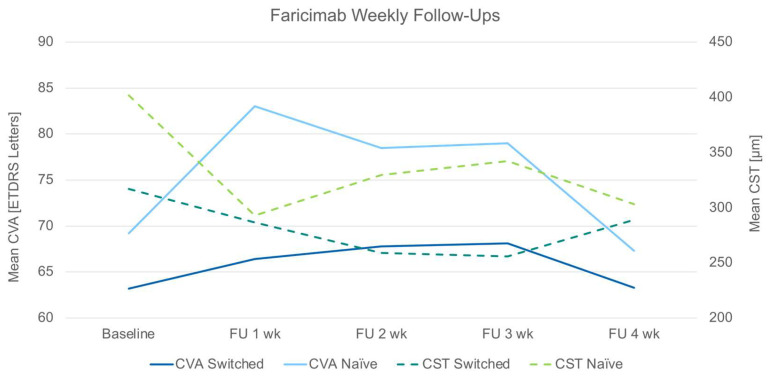
Graph plot of follow-ups (FU) for each week (wk) after first faricimab injection of corrected-visual acuity (CVA) of the switched group (dark-blue solid line), CVA of the naïve group (light-blue solid line), central subfield thickness (CST) of switched group (dark-green dashed line), CST of naïve group (light-green dashed line).

Switched patients showed a mean CVA increase of five letters on the ETDRS chart in week 2 after the first faricimab injection (Table 3, Figure 2). The CVA increase was maintained throughout week 3 and returned to the baseline level in week 4 without statistical significance (*p* = 0.86970) (Table 3, Figure 2). Statistical comparison was not calculated for the treatment-naïve subgroup due to its sample size of only six eyes.

The CST showed a decrease when comparing week 4 to baseline. This structural change was statistically significant for the switched group (*p* = 0.00383) (Table 3). Similar decreases were seen for the CSV (switched: *p* = 0.00702). The MNV flow area did decrease in absolute values in both groups when comparing the baseline with week 4. The difference was not statistically significant (switched: *p* = 0.13962) (Table 3). Four weeks after the first faricimab injection, no residual intra- (IRF) or subretinal fluid (SRF) could be detected within the macular OCT volume scan field in the switched group in 40% (dryness rate) and in the naïve group in 75% of the cases (Table 3). The switched group showed the strongest anatomical–structural response (CST, CSV, MNV flow area, dryness rate) around week 3 after the first faricimab injection (Table 3, Figure 2). The functional response (CVA) preceded the structural response by approximately one week (week 2) (Table 3, Figure 2).

## 4. Discussion

This study shows a functional and structural response to faricimab in nAMD patients under real-world conditions. Whilst significant structural changes were maintained throughout week 4 (*p* ≤ 0.05), the functional measures returned towards the baseline level 4 weeks after the first faricimab IVT (*p* ≥ 0.05). The switched group consisted of 61% MNV 1 lesions defined by multimodal imaging at study inclusion (Table 1), which was expected, as MNV type 1 is considered the most common MNV subtype in AMD [34,35]. Patients with MNV 1 are known to present with a better baseline CVA and to have a better long-term CVA outcome [36]. The same was visible in our dataset, providing a possible explanation as to why we did not see a statistically significant functional improvement after the first faricimab injection (plateau effect), whereas we could show a statistically significant treatment response in structural parameters (Table 3). Furthermore, the functional response due to the introduced Ang-2 inhibition might have been lagging behind the structural response. It is known that MNV 1 responds slower to anti-VEGF and needs more injections [35,37]. Both predominant types, MNV1 and MNV2, responded promptly and statistically significantly, which can be a promising sign when it comes to long-term visual acuity prognosis and disease control with longer intervals [38]. In contrast, both Leung et al. (structural: −25 µm, functional: −0.06 logMAR) [39] and the TRUCKEE study by Khanani et al. [40] could show a statistically significant functional improvement besides a statistically significant structural response. The TRUCKEE study showed the greatest functional (+4.9 letters) and structural (−84.5 µm) response after 4 weeks within their treatment-naïve subgroup [40]. These results are conclusive, as these eyes had not been exposed to either anti-VEGF-A or anti-Ang-2 before. The same trend in structural absolute values could be seen in the naïve subgroup of this study (−98.8 µm) (Table 3). However, the functional values did not improve (−1.9 letters) compared to the baseline (Table 3). The sample of the naïve group in this study was approximately 11% (6 vs. 57 eyes) of the size of the switched group (39 vs. 337; 12%), which is comparable to the TRUCKEE study. However, the absolute values of this study were smaller, not allowing for a valid statistical analysis. As most patients within the switched group had received intensive anti-VEGF pre-treatment (median 33 IVTs) (Table 1), a limited effect of the first faricimab IVT on the functional outcomes was expected (switched: *p* = 0.86970) (Table 3). A similar trend, with a statistically significant structural but not functional response, was found over a longer treatment and follow-up period (mean 6.98 months) by Ng et al. [41]. The structural response with a dryness rate of 40% in our cohort (Table 3) seemed promising, especially in a study population where a complete resolution of IRF and SRF could not be achieved with previous treatment regimes. This finding goes in line with the 39% dryness rate Ng et al. found in their cohort of 63 treatment-refractory nAMD eyes [41]. A lower rate of complete dryness of 24% was seen within the cohort of 190 treatment-refractive nAMD eyes that Leung et al. had evaluated over the mean course of 8.7 months [39]. The persistence of this effect over the course of several injections and the possibility to extend the treatment interval remain to be shown. If this will be the case, the prolonged suppression of Ang-2 levels compared to the VEGF-A levels might be one explanation [42]. Currently, there are no reports on real-world safety concerns with this new drug. Subsequent studies will evaluate if observed treatment effects persists over the course of several faricimab injections and if it allows for a significant extension of the treatment interval under real-world conditions. This might be a step further in slowing down the degenerative process, preserving residual visual function and lowering the treatment burden for nAMD patients.

In conclusion, faricimab showed a high safety profile during the tight weekly follow-up schedule. In our real-world cohort with mainly intensively anti-VEGF pre-treated patients, the first faricimab injection resulted in a fast structural and functional treatment response within the first two weeks after the injection. Structural treatment effects could be preserved throughout the first four weeks after treatment. The long-term efficacy of this novel drug under challenging real-world conditions must be evaluated by long-term follow-ups.

## Figures and Tables

**Table 1:** Demographic data.

	AMD Switched	AMD Treatment-Naïve
Number of eyes [*n*] (patients)	57 (48)	6 (5)
Age [years] median [Q1–Q3]	80 [74–86]	81 (78–89) [80–81]
Gender ratio of patients (female/male)	24 (50%):24 (50%)	2 (40%):3 (60%)
Eye ratio (OD:OS)	29 (51%):28 (49%)	3 (50%):3 (50%)
SE at baseline [dioptres] mean ± SD [95%CI]	−0.46 ± 1.40 [−0.83; −0.09]	1.06 ± 1.75 [−0.78; 2.90]
MNV type at baseline according to CONAN classification	MNV 1: 35 (61%) MNV 2 and mixed 1/2: 15 (25%) MNV 3 and any type 3 combination: 6 (11%) Not identifiable: 1 (2%)	MNV 1: 1 (17%) MNV 2 and mixed 1/2: 0 MNV 3 and any type 3 combination: 4 (67%) MNV mixed (1/2 or 1/3): 0 Not identifiable: 1 (17%)
Previous number of IVTs (median [Q1–Q3])	33 [14–53]	*N/A*
Previous IVT drugs (in alphabetical order)	aflibercept, bevacizumab, ranibizumab	*N/A*
Previous IVT interval (median [Q1–Q3])	4 [4–5]	*N/A*

Legend: 95%CI, 95% confidence interval; AMD, age-related macular degeneration; CONAN, Consensus on Neovascular AMD Nomenclature [32,33]; IOP, intraocular pressure; IQR, interquartile range defined as Q3−Q1; max, maximum; median, defined as second quartile (Q2, 50th percentile); IVT, intravitreal injection; mmHg, millimetre of mercury; MNV, macular neovascularisation; N/A, not applicable; NS, no support; Q1, first quartile (25th percentile); Q3, third quartile (75th percentile); OD, oculus dexter; OS, oculus sinister; SE, spherical equivalent defined as refraction sphere + (0.5 * refraction cylinder).

**Table 2:** Safety data.

	BL	FU 1wk	FU 2wk	FU 3wk	FU 4wk	T BL vs. FU 4wk
**AMD switched**						
IOP [mmHg] mean [95%CI]	13 ± 2.8 [12; 14]	12 ± 3.1 [11; 13]	12 ± 2.5 [11; 13]	13 ± 2.8 [12; 14]	13 ± 2.9 [12; 14]	* *p* < 0.0001
AC cells [no. of cells within 1 mm × 1 mm slit beam field]	0	0	1+ (1 eye)	0.5+ (1 eye)	0	N/A
AC flare	0	0	0	0	0	N/A
Vitreous cells [no. of cells within 1 mm × 1 mm slit beam field]	0	0	0	0	0	N/A
Retinal vessel status	OK	OK	OK	OK	OK	N/A
						
**AMD treatment-naïve**						
IOP [mmHg] at baseline mean [95%CI]	13 ± 1.3 [12; 15]	14 #	12 ± 2.3 #	12 ± 1.4 #	13 ± 1.3 [12; 14]	*p* = #
AC cells [no. of cells within 1 mm × 1 mm slit beam field]	0	0	0	0	0	N/A
AC flare	0	0	0	0	0	N/A
Vitreous cells [no. of cells within 1 mm x 1 mm slit beam field]	0	0	0	0	0	N/A
Retinal vessel status	0	0	0	0	0	N/A

Legend: *, statistically significant; #, too few data points to calculate statistical metrics; 95%CI, 95% confidence interval; AC, anterior chamber; AMD, age-related macular degeneration; BL, baseline visit; FU, follow-up visit; IOP, intraocular pressure; IQR, interquartile range defined as Q3−Q1; max, maximum; median, defined as second quartile (Q2, 50th percentile); IVT, intravitreal injection; min, minimum; mmHg, millimetre of mercury; N/A, not applicable; no., number; OD, oculus dexter; OS, oculus sinister; *p*, *p*-value defined as statistically significant when <0.05; Q1, first quartile (25th percentile); Q3, third quartile (75th percentile); SE, spherical equivalent defined as refraction sphere + (0.5 * refraction cylinder); T, one-sample, dependent, 2-tailed *t*-test; wk, week(s).

**Table 3:** Efficacy data.

AMD Switched	BL (*n* = 57)	FU 1wk (*n* = 27)	FU 2wk (*n* = 26)	FU 3wk (*n* = 25)	FU 4wk (*n* = 57)	BL vs. FU 4wk
CVA [correctly read ETDRS letters] mean [95% CI]	63.2 ± 18.5 [58.3; 68.1] MNV1: 64.9 ± 16.8 [59.2; 70.7] MNV2 and 1/2: 60.3 ± 23.8 [47.1; 73.4] MNV3 and any type 3: 65.8 ± 10.6 [54.7; 77.0]	66.4 ± 17.0 [60.2; 72.5] MNV1: 64.8 ± 19.4 [55.9; 73.6] MNV2 and 1/2: 71.9 ± 11.1 [62.6; 81.1] MNV3 and any type 3: 63.0 ± 11.5 [34.4; 91.6]	67.8 ± 18.1 [60.5; 75.1] MNV1: 64.1 ± 21.5 [52.6; 75.5] MNV2 and 1/2: 77.6 ± 3.4 [74.4; 80.7] MNV3 and any type 3: 65.0 ± 12.0 [35.2; 94.8]	68.1 ± 16.5 [61.3; 74.9] MNV1: 66.2 ± 19.1 [56.0; 62.6] MNV2 and 1/2: 75.3 ± 5.2 [69.8; 80.8] MNV3 and any type 3: 64.0 ± 15.9 [24.6; 103.4]	63.3 ± 19.7 [58.1; 68.5] MNV1: 65.4 ± 18.0 [59.2; 71.6] MNV2 and 1/2: 59.3 ± 24.7 [45.7; 73.0] MNV3 and any type 3: 66.0 ± 12.5 [52.9; 79.1]	∆ +0.1 W: *p* = 0.86970
CST [µm] mean [95% CI]	317.0 ± 78.6 [296.2; 337.8] MNV1: 315.9 ± 80.8 [288.1; 343.6] MNV2 and 1/2: 346.9 ± 69.9 [308.2; 385.7] MNV3 and any type 3: 268.5 ± 47.7 [218.5; 318.5]	286.4 ± 63.8 [263.4; 309.4] MNV1: 280.9 ± 54.7 [256.0; 305.8] MNV2 and 1/2: 318.8 ± 84.5 [248.1; 389.4] MNV3 and any type 3: 238.3 ± 8.3 [217.6; 259.0]	259.1 ± 47.4 [240.0; 278.3] MNV1: 259.5 ± 43.5 [236.3; 282.7] MNV2 and 1/2: 271.4 ± 62.6 [213.5; 329.3] MNV3 and any type 3: 228.3 ± 11.5 [199.8; 256.9]	255.7 ± 52.2 [234.2; 277.2] MNV1: 249.6 ± 29.4 [234.0; 265.3] MNV2 and 1/2: 286.8 ± 92.6 [189.7; 384.0] MNV3 and any type 3: 225.7 ±13.4 [192.3; 259.0]	289.0 ± 89.0 [265.4; 312.6] MNV1: 285.7 ± 89.0 [255.1; 316.3] MNV2 and 1/2: 325.3 ± 94.3 [273.1; 377.5] MNV3 and any type 3: 234.5 ±18.5 [215.1; 253.9]	∆ −27.9 W: * *p* = 0.00383
CSV [mm^3^] mean [95% CI]	0.249 ± 0.061 [0.233; 0.265] MNV1: 0.248 ± 0.062 [0.227; 0.269] MNV2 and 1/2: 0.273 ± 0.055 [0.243; 0.304] MNV3 and any type 3: 0.212 ± 0.038 [0.172; 0.252]	0.217 ± 0.047 [0.199; 0.236] MNV1: 0.206 ± 0.029 [0.191; 0.222] MNV2 and 1/2: 0.251 ± 0.065 [0.197; 0.306] MNV3 and any type 3: 0.187 ± 0.006 [0.172; 0.201]	0.202 ± 0.038 [0.186; 0.218] MNV1: 0.203 ± 0.035 [0.183; 0.222] MNV2 and 1/2: 0.210 ± 0.050 [0.164; 0.256] MNV3 and any type 3: 0.180 ± 0.010 [0.155; 0.205]	0.198 ± 0.037 [0.182; 0.214] MNV1: 0.196 ± 0.024 [0.183; 0.208] MNV2 and 1/2: 0.218 ± 0.070 [0.131; 0.305] MNV3 and any type 3: 0.177 ± 0.012 [0.148; 0.205]	0.228 ± 0.072; [0.209; 0.247] MNV1: 0.226 ± 0.074 [0.201; 0.252] MNV2 and 1/2: 0.255 ± 0.073 [0.215; 0.296] MNV3 and any type 3: 0.185 ± 0.014 [0.171; 0.199]	∆ −0.021 W: * *p* = 0.00702
MNV flow area [mm^2^] mean [95% CI]	3.07 ± 2.61 [2.09; 4.04] MNV1: 3.66 ± 2.81 [2.34; 4.97] MNV2 and 1/2: 2.36 ± 1.91 [0.59; 4.12] MNV3 and any type 3: 0.79 ± 0.27 [0.11; 1.47]	2.84 ± 2.62 [1.39; 4.29] MNV1: 2.98 ± 3.02 [0.81; 5.14] MNV2 and 1/2: 3.16 ± 1.44 [0.88; 5.45] MNV3 and any type 3: 0.16 ± #	2.62 ± 2.18 [1.46; 3.78] MNV1: 2.81 ± 2.57 [0.97; 4.65] MNV2 and 1/2: 2.30 ± 1.47 [0.75; 3.84] MNV3 and any type 3: #	2.62 ± 2.26 [1.36; 3.87] MNV1: 2.78 ± 2.62 [0.91; 4.66] MNV2 and 1/2: 2.28 ± 1.50 [0.43; 4.14] MNV3 and any type 3: #	2.83 ± 2.45 [1.64; 4.01] MNV1: 3.07 ± 2.86 [1.35; 4.80] MNV2 and 1/2: 2.29 ± 1.25 [0.98; 3.60] MNV3 and any type 3: #	∆ −0.24 W: *p* = 0.62916
No. eyes IRF No. eyes SRF No. eyes IRF + SRF No. eyes dry (dryness rate)	34/57 (60%) 19/57 (33%) 4/57 (7%) 0/57 (0%)	9/27 (33%) 7/27 (26%) 0 (0%) 11/27 (41%)	9/26 (35%) 2/26 (8%) 1/26 (4%) 14/26 (54%)	9/25 (36%) 2/25 (8%) 1/25 (4%) 13/25 (52%)	22/57 (39%) 9/57 (16%) 3/57 (5%) 23/57 (40%)	*N/A*
**AMD treatment-naïve**	**BL (*n* = 6)**	**FU 1wk (*n* = 2)**	**FU 2wk (*n* = 2)**	**FU 3wk (*n* = 2)**	**FU 4wk (*n* = 4)**	**BL vs. FU 4wk**
CVA [correctly read ETDRS letters] mean [95% CI]	69.2 ± 10.5 [58.2; 80.1] MNV1: 56.0 ± # MNV2 and 1/2: # MNV3 and any type 3: 73.3 ± 9.9 [57.4; 89.1]	83.0 ± # MNV1: # MNV2 and 1/2: # MNV3 and any type 3: 83.0 ± #	78.5 ± 7.8 [8.6; 148.4] MNV1: # MNV2 and 1/2: # MNV3 and any type 3: 78.5 ± 7.8 [8.6; 78.3]	79.0 ± 8.5 [2.8; 155.2] MNV1: # MNV2 and 1/2: # MNV3 and any type 3: 79.0 ± 8.5	67.3 ± 11.4 [55.4; 79.3] MNV1: 65.0 ± # MNV2 and 1/2: # MNV3 and any type 3: 66.5 ± 14.2 [43.8; 89.2]	∆ −1.9 W: #
CST [µm] mean [95% CI]	401.8 ± 121.3 [274.6; 529.1] MNV1: 509 # MNV2 and 1/2: # MNV3 and any type 3: 339.5 ± 93.6 [190.5; 488.5]	293.0 ± # MNV1: # MNV2 and 1/2: # MNV3 and any type 3: 293.0 ± #	329.5 ± 53.0 [−147.0; 806.0] MNV1: # MNV2 and 1/2: # MNV3 and any type 3: 329.5 ± 53.0	342.0 ± 79.2 [−369.6; 1053.6] MNV1: # MNV2 and 1/2: # MNV3 and any type 3: 342.0 ± 79.2 [−369.5; 1053.5]	303.0 ± 97.9 [200.3; 405.7] MNV1: 239.0 ± # MNV2 and 1/2: # MNV3 and any type 3: 323.5 ± 118.0 [135.7; 511.3]	∆ −98.8 W: #
CSV [mm^3^] mean [95% CI]	0.317 ± 0.095 [0.217; 0.416] MNV1: 0.400 # MNV2 and 1/2: # MNV3 and any type 3: 0.268 ± 0.072	0.230 ± # MNV1: # MNV2 and 1/2: # MNV3 and any type 3: 0.230 ± #	0.260 ± 0.042 [−0.121; 0.641] MNV1: # MNV2 and 1/2: # MNV3 and any type 3: 0.260 ± 0.042	0.265 ± 0.064 [−0.307; 0.837] MNV1: # MNV2 and 1/2: # MNV3 and any type 3: 0.265 ± 0.064 [−0.307; 0.837]	0.260 ± 0.088 [0.121; 0.399] MNV1: 3 MNV2 and 1/2: # MNV3 and any type 3: 0.273 ± 0.102 [0.020; 0.527]	∆ −0.057 W: #
MNV flow area [mm^2^] mean [95% CI]	1.75 ± # MNV1: # MNV2 and 1/2: # MNV3 and any type 3: 1.75 ± #	2.16 ± 0.66 [−3.77; 8.09] MNV1: # MNV2 and 1/2: # MNV3 and any type 3: 2.16 ± 0.66 [−3.77; 8.09]	2.80 ± # MNV1: # MNV2 and 1/2: # MNV3 and any type 3: 2.80 ± #	1.69 ± # MNV1: # MNV2 and 1/2: # MNV3 and any type 3: 1.69 ± #	0.61 ± # MNV1: # MNV2 and 1/2: # MNV3 and any type 3: 0.61 ± #	∆ −1.14 W: #
No. eyes IRF No. eyes SRF No. eyes IRF + SRF No. eyes dry (dryness rate)	5/6 (83%) 1/6 (17%) 0/6 (0%) 0/6 (0%)	1/1 (100%) 0/1 (0%) 0/1 (0%) 0/1 (0%)	1/2 (50%) 0/2 (0%) 0/2 (0%) 1/2 (50%)	1/2 (50%) 0/2 (0%) 0/2 (0%) 1/2 (50%)	1/4 (25%) 0/4 (0%) 0/4 (0%) 3/4 (75%)	*N/A*

Legend: *, statistically significant; #, too few data points to calculate statistical metrics; 95%CI, 95% confidence interval; AMD, age-related macular degeneration; BL, baseline visit; CST, central subfield thickness in OCT of 1 mm circle of ETDRS grid; CSV, central subfield volume in OCT of 1 mm circle of ETDRS grid; ∆, delta, difference of values; ETDRS, Early Treatment of Diabetic Retinopathy Study; FU, follow-up visit; MNV, macular neovascularisation; n, number of eyes included; N/A, not applicable; no., number; *p*, *p*-value defined as statistically significant when <0.05; W, two-tailed Wilcoxon signed-rank test; wk, week(s).

## Data Availability

The data, aside from the data published in this manuscript, are not publicly available due to privacy restrictions.
